# Influence of development, postharvest handling, and storage conditions on the carbohydrate components of sweetpotato (*Ipomea batatas Lam*.) roots

**DOI:** 10.1002/fsn3.496

**Published:** 2017-08-27

**Authors:** Agnes Nabubuya, Agnes Namutebi, Yusuf Byaruhanga, Judith Narvhus, Trude Wicklund

**Affiliations:** ^1^ Department of Food Technology and Nutrition School of Food Technology Nutrition and Bio‐Engineering Makerere University Kampala Uganda; ^2^ Department of Chemistry, Biotechnology and Food Science Norwegian University of Life Sciences Ås Norway

**Keywords:** pasting properties, postharvest handling, storage, sugars, sweetpotato, total starch

## Abstract

Changes in total starch and reducing sugar content in five sweetpotato varieties were investigated weekly during root development and following subjection of the roots to different postharvest handling and storage conditions. Freshly harvested (noncured) roots and cured roots (spread under the sun for 4 days at 29–31°C and 63–65% relative humidity [RH]) were separately stored at ambient conditions (23°C–26°C and 70–80% RH) and in a semiunderground pit (19–21°C and 90–95% RH). Changes in pasting properties of flour from sweetpotato roots during storage were analyzed at 14‐day intervals. Significant varietal differences (*p* < .05) in total starch, sucrose, glucose, maltose, and fructose concentrations were registered. The total starch and sucrose content of the roots did not change significantly (*p* < .05) during root development (72.4 and 7.4%, respectively), whereas the average concentrations of glucose, maltose, and fructose decreased markedly (0.46–0.18%, 0.55–0.28%, and 0.43–0.21%), respectively. Storage led to decrease in total starch content (73–47.7%) and increase in sucrose and glucose concentrations (8.1–11.2% and 0.22–1.57%, respectively). Storage also resulted in reduction in sweetpotato flour pasting viscosities. Curing resulted in increased sucrose and glucose concentrations (9.1–11.2% and 0.45–0.85%, respectively) and marked reduction (*p* < .05) in total starch content (72.9–47.6%). This resulted in low pasting viscosities compared to flour from storage of uncured roots. These findings show that significant changes occur in the carbohydrate components of sweetpotato roots during storage compared to development and present an opportunity for diverse utilization of flours from sweetpotato roots in the food industry.

## 1 INTRODUCTION

Sweetpotato roots are very important staples in many parts of the world, especially in the tropics. The roots exist in different varieties, which vary in skin and flesh and color. Sweetpotato roots contain high amounts of carbohydrates; 80–90% of dry weight Lebot, 2009; Woolfe (1992), most of which is starch (50–80%) of the dry matter. The roots also contain varying amounts of sugars (Nabubuya, Namuteb, Byaruhanga, Narvhus, & Wicklund, 2012; Woolfe 1992), depending on cultivar, production environment, or an interaction of the two conditions, Lewis, Lancester, Meredith, & Walter, 2010; Takahata, Noda, and Sato (1995). Free sugars and native starch in sweetpotato roots have been shown to have considerable impact on both the eating quality and processing traits (Huang, Picha, Kilili, & Johnson, 1999; Takahata et al., 1995). Storage of sweetpotato roots has been shown to result in changes in the roots carbohydrate components: decreasing starch and increasing sugar contents, especially reducing sugars (Morrison et al., 1993; Takahata et al., 1995). The changes in carbohydrate fractions (starch and sugar contents of sweetpotato roots) during storage are attributed to the activities of endogenous amylolytic enzymes (Morrison et al. 1993; Takahata et al., 1995; Walter, Purcell, & Nelson, 1975). Amylase enzymes hydrolyze the glycosidic bonds in the starch granule, yielding simpler sugars (van der Maarel, van der Veen, & Uitdehaag, 2002). It has also been reported that changes occur in the carbohydrate content; starch and sugars of sweetpotato roots during development (Bonte & Picha, 2000; Wang, Lee, Chen, Huang, & Su, 2000).

In Sub‐Saharan Africa, a variety of postharvest handling conditions and storage methods have been employed in order to enhance the shelf life of harvested sweetpotato root crops. The methods include curing of root by spreading in the sun, to allow for root skin to hardening and wound healing (Leonard & Louis, 1955). Alternatively, sweetpotato roots are left in the farm land ground and harvested piece meal as required (Smit, 1997). The storage methods in use on the other hand include: pit stores Moyo et al. (2004), ambient conditions, and in sacks. While there is considerable documentation on the changes in sweetpotato starch and sugars during storage, it is not very clear how the different postharvest handling conditions and storage methods used in Sub‐Saharan Africa impact on the changes in the carbohydrate components of the sweetpotato roots. There are also conflicting reports about the variations in roots and tuber flour pasting properties during storage (Golachowski, 1985; Ridley & Hogan, 1976).

This study therefore presents metabolic changes in the carbohydrate components during development and resulting from typical Sub‐Saharan postharvest handling and storage conditions of sweetpotato roots of selected Ugandan varieties. Secondly, the magnitude of these changes on the carbohydrate components impacting the subsequent industrial use of the roots is presented. This work builds on an earlier study on the behavior of endogenous amylases of sweetpotato roots during development and storage.

## 2 MATERIALS AND METHODS

### 2.1 Sweetpotato materials

Five sweetpotato varieties NASPOT 9, NASPOT 10, Kakamega*,* NASPOT 1, and NASPOT 2 used in this study were cultivated in three replicate plots in an experimental field at the National Agriculture Crop Resource Research Institute (NACCRI) in Uganda. These sweetpotato varieties were chosen basing on previous work on their variation in chemical composition Nabubuya, Namuteb, Byaruhanga, Narvhus, & Wicklund (2012) and amylase activity during storage (Nabubuya, Namutebi, Byaruhanga, Narvhus, Stenstrøm, et al., 2012). Sampling of developing roots began 10 weeks after planting, with an average root weight of 50 g and harvesting was done at intervals of 1 week for all the varieties. At the fifth sampling time, which corresponded to mature harvest time (14 weeks), roots from all varieties were harvested for storage. The roots were handled in two ways prior to storage; freshly harvested roots were either stored directly (noncured) or they were cured by initially spreading under the sun for 4 days (29–31°C and 63–65% RH). The roots were then subjected to two storage conditions; ambient/room storage (23–26°C and 70–80% RH) or pit storage (19–21°C and 90–95% RH). The pit store was a 60‐cm pit, lined with spear grass (*Imperata cylindrica*). The roots were stored for 8 weeks and analyzed weekly for changes in total starch, sucrose, glucose, fructose, and maltose and after every 2 weeks for flour pasting properties.

### 2.2 Sample preparation for laboratory analysis

Four sound roots were randomly selected for each sweetpotato variety from each of the three replicates to make composite samples for subsequent analyses. For developing sweetpotatoes, the weight of the individual roots increased from 50 to 180 g over the study period and the mean weight for roots in storage was 200 g. Each of the selected roots was washed under running water, peeled, halved longitudinally, and uniformly grated. The grated tissue from the four roots per replicate was combined and mixed thoroughly. Samples for total starch and sugar analysis were prepared by freeze drying grated root tissue for 24 hr and milling it into flour using a laboratory mill (3303‐ Falling number, Huddings, Sweden). While flour for pasting properties was prepared by oven drying grated sweetpotato tissue at 45°C for 16 hr (Gallenkamp, UK), milled using a laboratory mill (Wondermill, model 70, Korea) and sieved through a 250 μm mesh.

### 2.3 Methods

#### 2.3.1 Reagents

The reagents used were of analytical grade and were obtained from Megazyme International Ireland Ltd., Bray, C. Wicklow and Sigma‐Aldrich Chemical Company**.**

### 2.4 Sugar analysis

Quantification of individual sugars was a modification of the analysis described by Knudsen (1997). Samples (1.0 g) were extracted with 40‐ml ethanol—MilliQ water (1:3 v/v)—for 24 hr during which the extract was mixed using an electric mixer for 30 min. The extract was centrifuged at 2200 ***g*** for 30 min before 2 ml of an internal standard (arabinose, 1 mg ml^−1^) was added to 4 ml of the extract. The extract was purified using C18 cartridges (Water Corporation, Milford, MA), which had been washed with 2 ml of methanol and 5 ml of MilliQ water. It was further filtered through a 0.2 μm filter (Pall Life Sciences, 600 South Wagner Rd), and vacuum dried at 50°C (Vortex‐Evaporator, H. Haake Buchler Product, Saddle Brook, NJ). The dried sample was mixed in 110 μl of MilliQ water and filtered using a Millex‐GV filter (13 mm, 0.22 μm). Exactly 20 μl of the filtrate was used to determine the concentration of sucrose, maltose, glucose, and fructose using HPLC. The HPLC system used consisted of a Perkin Elmer series 410 delivery pump, series 200 refractive index detector, series 200 injector valve (Burnsville, MN), and an Aminex HPX‐87H, 300 × 7.8 mm id column (Macherey Nagel, UK). MilliQ water was used as the mobile phase, at a flow rate of 0.4 ml min^−1^, and the column temperature was maintained at 80°C. External standard solutions of sucrose, maltose, glucose, and fructose (Sigma Chemical Co.) were used for calibration, identification, and quantification of the respective sugars.

### 2.5 Total starch determination

The total starch content in the sweetpotato flours was determined using the amyloglucosidase/α‐amylase method (McCleary & Monaghan, 2002) which involved two phases; partial hydrolysis followed by solubilization of starch in the flour by α‐amylase and quantitative hydrolysis of dextrins to glucose by amyloglucosidase. Sweetpotato flour (100 mg) was dispersed in 0.2 ml of 80% ethanol and immediately 3 ml of thermostable α‐amylase mixed with 100 mmol L^−1^ sodium acetate buffer (pH 5.0) was added and heated in a boiling water bath for 6 min. It was then placed in a water bath at 50°C and 0.1 ml of amyloglucosidase added then incubated for 30 min. Three mililiter of glucose determination reagent (GOPOD‐containing GOPOD reagent buffer and GOPOD reagent enzymes) was added to 0.1 ml of supernatant after centrifuging at 3,000 ***g*** for 10 min. The above mixture was incubated at 50°C for 20 min and the absorbance read at 510 nm against a reagent blank. Regular maize starch (supplied by Megazyme) was used to standardize the procedure.

### 2.6 Flour pasting properties

A rapid viscoanalyzer (RVA, model 4, Newport Scientific, Warriewood, Australia) with Thermocline software was used to evaluate the pasting properties of sweetpotato flour. The tests were conducted following standard pasting profile, standard 1. Sweetpotato flour (3.5 g in 25 ml of water) adjusted to 14% moisture content was subjected to a controlled heating and cooling regime under constant shear in the RVA. The time–temperature regime of the equipment was as follows: The slurry was stirred at 960 rpm for 1 min and at 160 rpm for the rest of the test time. The temperature was increased from 50 to 95°C in 4 min at which it was held for 3 min and subsequently cooled to 50°C in 4 min. This was followed by a period of 1 min where the temperature was kept at 50°C. The flour pasting test process lasted for 13 min. The RVA parameters of interest included peak viscosity, holding strength (trough), and final viscosity. The viscosity was expressed in centipoises (cP).

### 2.7 Statistical analysis

The data were subjected to ANOVA (general linear model) using Minitab (Minitab inc., State College, PA) version 16 and the means were separated using Tukey's test. Significance was accepted at *p* < .05. The results are presented as means with respective standard deviations. The experiment was done in triplicate.

## 3 RESULTS AND DISCUSSION

### 3.1 Changes in sugars and starch contents during root development

**Figure 1:**
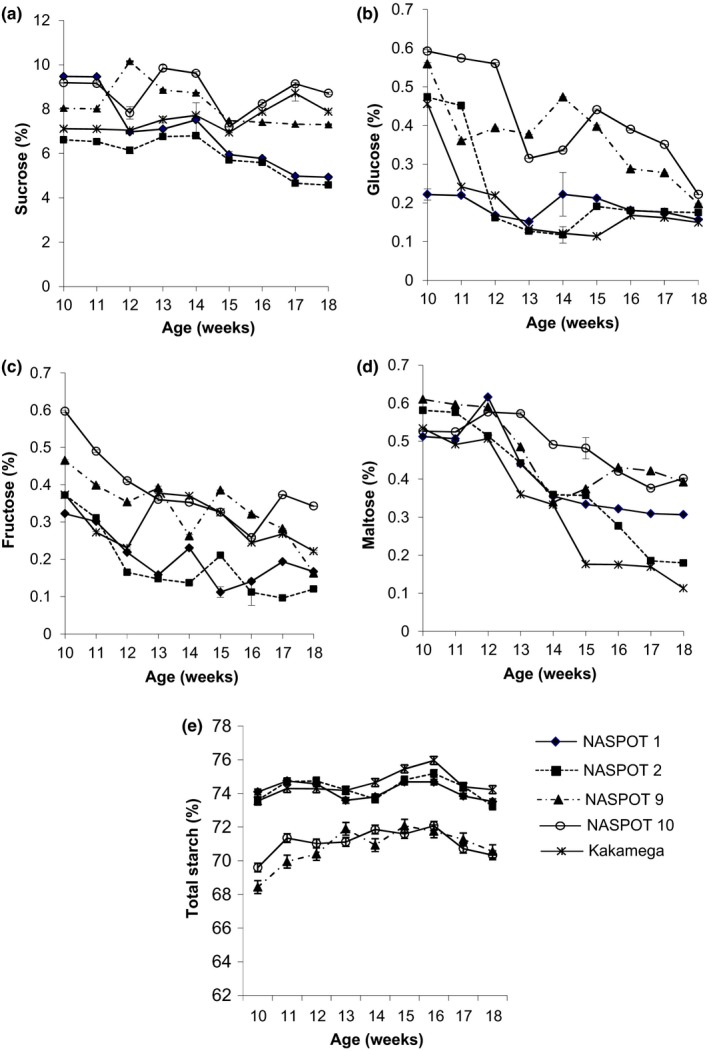
Carbohydrate content of five sweetpotato varieties during root development (% DM) (a) Sucrose, (b) Glucose, (c) Maltose, (d) Fructose, and (e) Total starch

Sucrose was the major sugar in all the sweetpotato varieties, which fluctuated during root development, although starch content at the start (10th week) was not significantly different (*p* > .05) from the 18th week, the end (Figure 1). Sucrose content varied significantly (*p* < .05) among varieties with NASPOT 10 having the highest (8.5%) and NASPOT 2 the lowest content, 5.9% (Figure 1a). Our findings differed from Bonte and Picha (2000) who found a consistent increase in sucrose content (56%) in sweetpotato roots during development. Sucrose that accumulates during root development is used to supply the demands for structural and storage carbohydrates and to facilitate the respiratory pathway (ap Rees & Morrell, 1990), thus leading to fluctuation in content. Glucose, fructose, and maltose contents in sweetpotato roots also varied significantly (*p* < .05) among varieties, with NASPOT 10 consistently having the highest contents, 0.42, 0.39, and 0.49%, respectively, (Figure 1b,c,d). Although these sugars fluctuated during development, a decrease was registered by the 18th week. NASPOT 9 and NASPOT 10 registered about 50% reduction in glucose concentration which concurred with Bonte and Picha (2000) six sweetpotato varieties and Lewis et al. (2010) potato varieties studied. The results obtained in the study are also in line with other results which showed differences in individual sugars among sweetpotato varieties (Woolfe, 1992).

Significant differences in starch contents (*p* < .05) among varieties were registered with Kakamega (74.5%) having the highest and NASPOT 9 the lowest content (70.8%) by the 18th week (Figure 1e). NASPOT 9 and NASPOT 10 had consistently the lowest starch contents. Starch in the sweetpotato roots increased slightly but not significantly (*p* > .05) by the 12th week and thereafter fluctuated. Starch content in all the varieties by the 18th week was not significantly different (*p* > .05) from the 10th week, which concurred with Lewis et al. (2010) study on developing *Solanum tuberosum* tuber. Starch is continually both deposited and degraded during root development due to the activity of both biosynthetic and degrading enzymes (Isherwood, 1973). Concentration of starch at any one time is a result of the balance of the activities of these enzymes.

### 3.2 Changes in sweetpotato root starch and sugars during root storage

#### 3.2.1 Sugars

**Figure 2:**
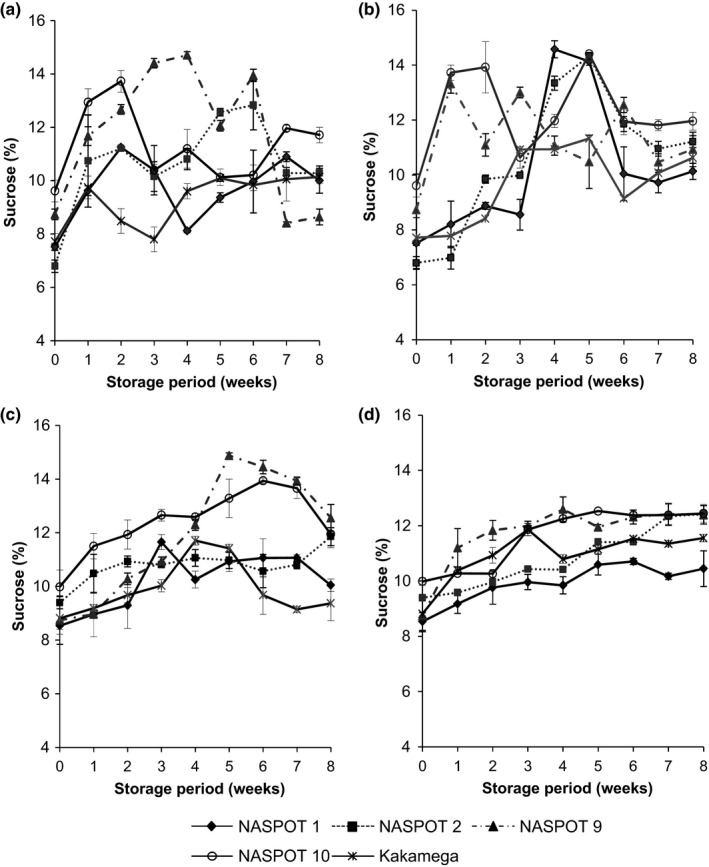
Changes in sucrose concentration (% DM) of five sweetpotato varieties subjected to different postharvest handling and storage conditions: (a) Fresh (uncured) roots stored under room conditions (23–26°C and 70–80% RH), (b) Fresh roots stored in the pit (19–21°C and 90–95% RH), (c) Cured roots stored under room conditions (23–26°C and 70–80% RH), and (d) Cured roots stored in the pit (19–21°C and 90–95% RH)

**Figure 3:**
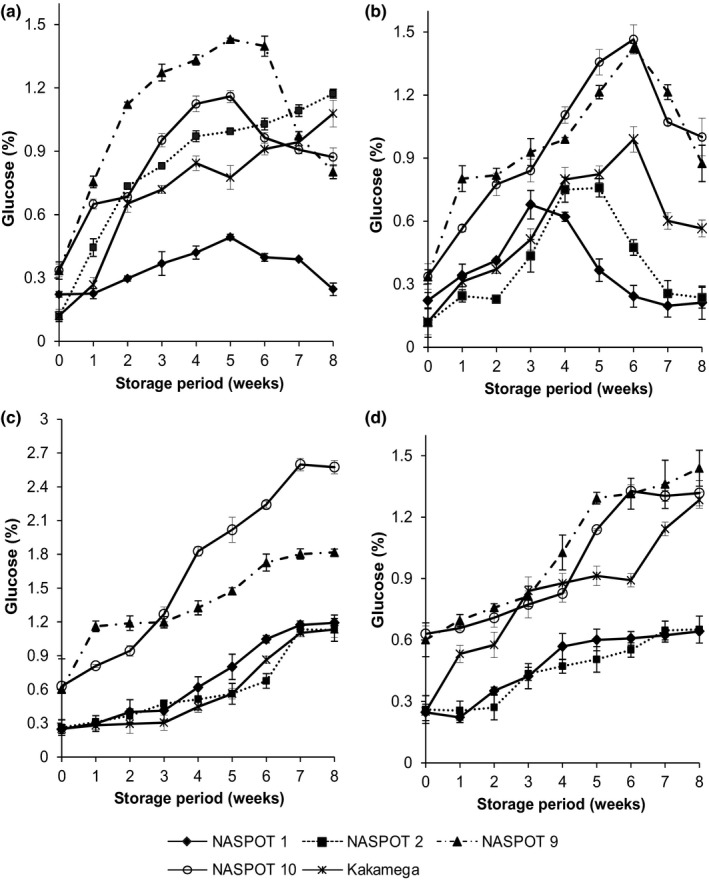
Changes in glucose concentration (% DM) of five sweetpotato varieties subjected to different postharvest handling and storage conditions: (a) Fresh roots stored under room conditions (23–26°C and 70–80% RH), (b) Fresh roots stored in the pit (19–21°C and 90–95% RH), (c) Cured roots stored under room conditions (23–26°C and 70–80% RH), and (d) Cured roots stored in the pit (19–21°C and 90–95% RH)

A significant variation (*p* < .05) in the different sugars was recorded among varieties during the storage period (Figures 2 and 3). There was slight increase in the sucrose content (Figure 2), although the different varieties accumulated sucrose differently. Whereas there was almost a consistent increase in sucrose content in cured roots, the changes in noncured roots were inconsistent, showing sharp fluctuations (Figure 2). There is no clear explanation for the fluctuation of sucrose content in the uncured roots, although some other studies have shown similar trends during storage of sweetpotatoes (Takahata et al., 1995).

The glucose content in the sweetpotato roots also increased during storage (Figure 3). The increase was consistent in cured roots, but reached maximum levels in weeks 5 and 6 in the noncured roots, then decreased (Figure 3a,b). NASPOT 9 and NASPOT 10 consistently had the highest glucose levels at the start of the study and the starting values were higher in cured roots (Figure 3c,d). NASPOT 9 and NASPOT 10 also displayed higher glucose content than the other varieties throughout the storage. Cured NASPOT 10 contained significantly higher glucose levels than other varieties especially during room storage. Kakamega displayed its highest and lowest glucose levels in the cured form in the pit and room, respectively**.**

Zhang, Wheatley, and Corke (2002) reported a similar trend in sugars in stored sweetpotato roots, although it was noted that sugars did not increase further after 60 days in storage. Morrison et al. (1993) on the other hand suggested that changes in individual and total sugar concentrations for sweetpotato lines (varieties) were relatively minor during storage. Takahata et al. (1995) reported a sharp increase in sweetpotato sucrose content, but negligible changes in glucose and fructose concentrations. Results from our study on the other hand showed minor variation in sucrose, but significant changes in glucose concentration especially in cured roots. Curing of sweetpotato roots results in increased sugar content due to increased breakdown of starch (Edmunds et al., 2008). The increase in the sweetpotato sucrose content during storage could be attributed to a number of factors related to its metabolism. The sucrose metabolism is, however, not well understood as a number of enzymes are believed to cause its accumulation during storage (Takahata et al., 1995). It could be as a result of the hydrolytic action of amylases on starch or the action of sucrose synthetase (Takahata et al., 1995).

#### 3.2.2 Starch

**Figure 4:**
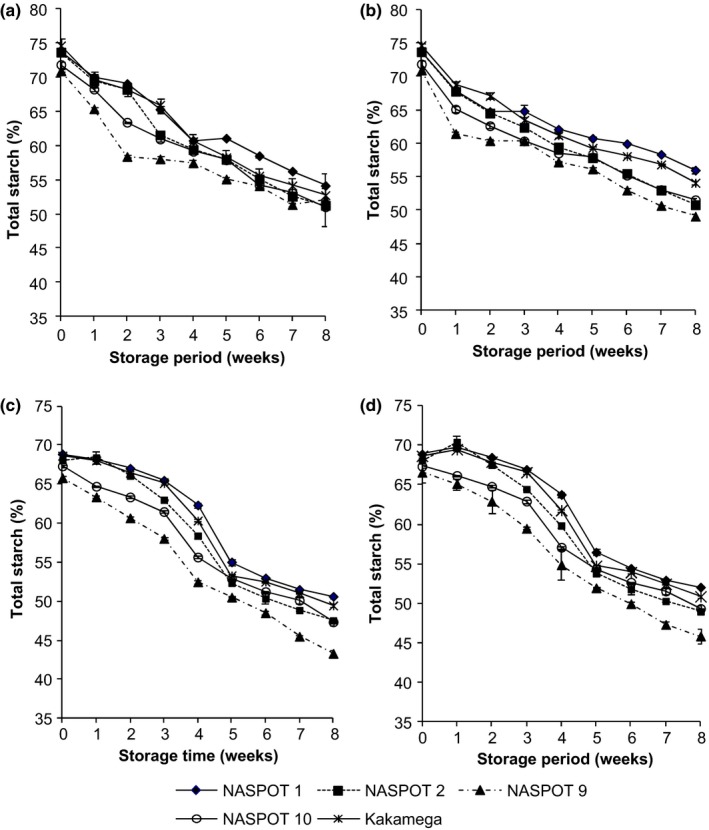
Changes in total starch content (% DM) of five sweetpotato varieties subjected to different postharvest handling and storage conditions: (a) Fresh roots stored under room conditions (23–26°C and 70–80% relative humidity), (b) Fresh roots stored in the pit (19–21°C and 90–95% RH), (c) Cured roots stored under room conditions (23–26°C and 70–80% RH), and (d) Cured roots stored in the pit (19–21°C and 90–95% RH)

There was significant variation (*p* < .05) in total starch content among the sweetpotato varieties during storage with NASPOT 1 and NASPOT 9 having the highest and lowest starch content, respectively, in all storage conditions (Figure 4). The total starch content decreased significantly (*p* < .05) in all sweetpotato varieties during storage. Curing led to significantly lower final total starch content (47.7%) than in noncured roots (52.3%). Decrease in sweetpotato root total starch content during storage is reported to be a result of the activity of root enzymes especially amylases (Walter et al., 1975). Amylase activity in sweetpotato roots increases during storage and is reported to have a significant role in decreasing starch during storage or sprouting (Deobald, Hasling, & Catalano, 1971; Morrison et al., 1993; Nabubuya, Namutebi, Byaruhanga, Narvhus, Stenstrøm, et al., 2012). Nabubuya, Namutebi, Byaruhanga, Narvhus, Stenstrøm, et al. (2012) also reported variation in amylase activity with different postharvest handling and storage conditions.

### 3.3 Flour pasting properties

All the pasting viscosity parameters (peak, trough, and final viscosities) of flours from the sweetpotato roots varied significantly (*p* < .05) among varieties and with postharvest handling and storage conditions (Tables 1, 2, and 3). Although storage generally caused significant reduction in the peak, trough, and final viscosities of the sweetpotato flours (2504 to 768, 1064 to 23, and 1640 to −15 cP, respectively), curing of the roots led to significantly (*p* < .05) lower viscosities than those observed in uncured roots (Tables 1, 2, and 3). The results also revealed that curing of sweetpotato roots led to faster reduction in flour peak viscosities to the extent that it took four more weeks for flours from noncured roots to attain the same values (Table 1). There is generally scanty documentation on changes in pasting viscosities during storage, although Zhang et al. (2002) observed slight reduction in all pasting viscosities of sweetpotato flour after 60 days in storage. Our results, however, showed drastic reductions in the pasting viscosities during storage especially for cured roots with changes in the viscosities being noticeable even in the second week, especially in the trough and final viscosities (421 to 30 and 403 to 30 cP, respectively, Tables 2 and 3). The differences observed between these results and the previous could be due to differences in varieties, environmental and postharvest handling, and storage conditions. Akinwande, Adeyemi, Maziya, and Asiedu (2007) also reported reduction in pasting viscosities of yam (*Dioscorea rotundata*) starch during storage. Conflicting results have, however, been reported from *Solanum tuberosum,* where Golachowski (1985) reported increase in viscosity, but Ridley and Hogan (1976) observed that storage led to a decrease.

**Table 1:** Peak viscosities (cP) of sweetpotato pastes as affected by postharvest handling and storage conditions of sweetpotato roots

Sweetpotato variety	Postharvest handling condition	Storage duration				
		Day 1	Day 14	Day 28	Day 42	Day 56
NASPOT 1	RF	2504^a^	1072^b^	945^a^	795^b^	768^a^
	RC	1212^b^	551^d^	432^b^	302^c^	272^c^
	PF	2504^a^	1259^a^	991^a^	921^a^	714^b^
	PC	1212^b^	784^c^	435^b^	315^c^	285^c^
NASPOT 2	RF	1012^a^	652^b^	527^b^	290^b^	252^b^
	RC	538^b^	356^c^	102^c^	81^c^	42^d^
	PF	1012^a^	995^a^	553^a^	513^a^	424^a^
	PC	538^b^	359^c^	135^c^	93^c^	67^c^
NASPOT 9	RF	933^a^	344^b^	233^b^	101^a^	73^d^
	RC	544^b^	120^c^	34^d^	40^c^	25^c^
	PF	933^a^	537^a^	568^a^	480^a^	360^a^
	PC	544^b^	142^c^	55^c^	58^c^	33^c^
NASPOT 10	RF	1450^a^	774^a^	310^b^	147^b^	126^b^
	RC	726^b^	265^c^	85^c^	56^c^	36^c^
	PF	1450^a^	564^b^	495^a^	484^a^	425^a^
	PC	726^b^	217^c^	89^c^	73^c^	45^c^
Kakamega	RF	1823^a^	1323^a^	720^b^	514^b^	465^b^
	RC	1429^b^	698^b^	403^c^	95^c^	59^c^
	PF	1823^a^	1182^a^	1026^a^	655^a^	630^a^
	PC	1429^b^	805^b^	238^d^	93^c^	63^c^

RF, Room fresh (23–26°C and 70–80% RH); RC, Room cured (29–31°C and 63–65% RH for 4 days then at 23–26°C and 70–80% RH); PF, Pit fresh (19–21°C and 90–95% RH); PC, Pit cured (29–31°C and 63–65% RH for 4 days then at 19–21°C and 90–95% RH).

Data are presented as means of three replicates. Values followed by the same superscript letters are not significantly different among storage conditions within each variety (*p* < .05).

**Table 2:** Trough viscosities (cP) of sweetpotato pastes as affected by postharvest handling and storage conditions of sweetpotato roots

Sweetpotato variety	Postharvest handling condition	Storage duration				
		Day 1	Day 14	Day 28	Day 42	Day 56
NASPOT 1	RF	1064^a^	397^b^	140^b^	33^b^	23^b^
	RC	577^b^	−16^d^	−29^d^	−8.7^c^	−13^c^
	PF	1064^a^	484^a^	303^a^	234^a^	190^a^
	PC	577^b^	126^c^	7^c^	−29^d^	−41^d^
NASPOT 2	RF	611^a^	92^b^	19^a^	−34^c^	−46^c^
	RC	407^b^	7^c^	−40^b^	−24^b^	−38^b^
	PF	611^a^	348^a^	33^a^	15^a^	7^a^
	PC	407^b^	−10^c^	−28^b^	−40^c^	−48^c^
NASPOT 9	RF	538^a^	1.6^b^	−26^b^	−41^a^	−56^b^
	RC	55^b^	−8^c^	−37^c^	−57^c^	−73^c^
	PF	538^a^	25^a^	26^a^	11^a^	4^a^
	PC	55^b^	−14^c^	−35^c^	−37^b^	−75^c^
NASPOT 10	RF	845^a^	223^a^	−37^c^	−51 ± 2^c^	−64^c^
	RC	228^b^	−24^d^	−34^c^	−32 ± 8^b^	−44^b^
	PF	845^a^	228^a^	36^a^	15 ± 25^a^	8^a^
	PC	228^b^	−5^c^	−8^b^	−34 ± 4^b^	−38^b^
Kakamega	RF	870^a^	301^b^	23^b^	15^b^	−67^a^
	RC	638^b^	−33^d^	−34^c^	−53^d^	−49^b^
	PF	870^a^	517^a^	142^a^	75^a^	53^a^
	PC	638^b^	14^c^	−17^c^	−25^c^	−56^bc^

RF, Room fresh (23–26°C and 70–80% RH); RC, Room cured (29–31°C and 63–65% RH for 4 days then at 23–26°C and 70–80% RH); PF, Pit fresh (19–21°C and 90–95% RH); PC, Pit cured (29–31°C and 63–65% RH for 4 days then at 19–21°C and 90–95% RH).

Data are presented as means of three replicates. Values followed by the same superscript letters are not significantly different among storage conditions within each variety (*p* < .05).

**Table 3:** Final viscosities (cP) of sweetpotato pastes as affected by postharvest handling and storage conditions of sweetpotato roots

Sweetpotato variety	Postharvest handling condition	Storage period				
		Day 1	Day 14	Day 28	Day 42	Day 56
NASPOT 1	RF	1640^a^	581^a^	220^b^	24^b^	−15^b^
	RC	769^b^	16^d^	−5^d^	−17^d^	−34^c^
	PF	1640^a^	716^a^	407^a^	270^a^	195^a^
	PC	769^b^	165^c^	13^c^	−5^c^	−25^bc^
NASPOT 2	RF	901^a^	144^b^	32^b^	−8^b^	−38^b^
	RC	66^b^	−8^c^	−42^c^	−30^c^	−44^c^
	PF	901^a^	524^a^	58^a^	20^a^	6^a^
	PC	66^b^	−14^c^	−22^c^	−28^c^	−42^c^
NASPOT 9	RF	687^a^	−3^b^	−32^b^	−40^c^	−52^b^
	RC	51^b^	−24^c^	−47^c^	−57^c^	−73^c^
	PF	687^a^	51^a^	48^a^	26^a^	11^a^
	PC	51^b^	−25^c^	−37^b^	−21^b^	−71^c^
NASPOT 10	RF	1445^a^	356^a^	−10^b^	−54^c^	−69^b^
	RC	325^b^	−40^d^	−27^c^	−33^b^	−51^b^
	PF	1445^a^	322^a^	32^a^	19^a^	13^a^
	PC	325^b^	−12^c^	−23^c^	−42^bc^	−56^b^
Kakamega	RF	1357^a^	445^b^	69^b^	−18 ± 4^b^	−32^c^
	RC	805^b^	−14^d^	−22^c^	−51 ± 15^d^	−59^c^
	PF	1357^a^	763^a^	361^a^	181 ± 3^a^	135^a^
	PC	805^b^	43^c^	−12^c^	−34 ± 4^b^	−47^bc^

RF, Room fresh (23–26°C and 70–80% RH); RC, Room cured (29–31°C and 63–65% RH for 4 days then at 23–26°C and 70–80% RH); PF, Pit fresh (19–21°C and 90–95% RH); PC, Pit cured (29–31°C and 63–65% RH for 4 days then at 19–21°C and 90–95% RH).

Data are presented as means of three replicates. Values followed by the same superscript letters are not significantly different among storage conditions within each variety (*p* < .05).

Starch is the main component of sweetpotato roots, decrease in starch during storage would affect the pasting viscosities of sweetpotato pastes (Zhang et al., 2002). The low pasting viscosities of the sweetpotato pastes obtained in this study could be attributed to the reduction in native starch content and increases in reducing sugar content during storage. Our results showed a sharp decrease in the pasting viscosities of sweetpotato pastes, with slight hydrolysis of starch in the first 2 weeks of storage. The reduction was, however, gradual in subsequent weeks. Approximately 5–10% reduction in starch led to 50% decrease in peak viscosity in the first 2 weeks in all varieties and 75% reduction in trough and final viscosities especially in cured roots. Curing causes marked increase in amylase activity Nabubuya, Namutebi, Byaruhanga, Narvhus, Stenstrøm, et al. (2012), which in turn reduces the native starch content and increases in low molecular weight starch in sweetpotato roots (Boyette, Estes, Rubin, & Sorensen, 1997). The starch which has been acted upon by amylase enzymes has reduced swelling ability during pasting, hence the low viscosities observed in this study (Noda et al., 2004). Other factors such as protein and lipid content of flours and their pH (not investigated in this study) have also been reported to influence flour pasting properties (Walker, Ross, Wrigley, & McMaster, 1988). Results from this study revealed trends in sweetpotato root total starch content, individual sugars, and flour pasting properties during storage similar to those from previous studies (Morrison et al., 1993a, 1993b; Zhang et al., 2002) regardless of the differences in postharvest handling and storage conditions (temperatures and relative humidity) used in this study.

### 3.4 Functional implications of sweetpotato roots in development and storage

The functionality of sweetpotato roots is highly dependent on the endogenous amylases (Lilia & Harold, 1999), which impact on the starch and reducing sugar contents of the roots (Morrison et al., 1993a, 1993b). According to Nabubuya, Namutebi, Byaruhanga, Narvhus, Stenstrøm, et al. (2012) sweetpotato roots attain maximum amylase activity at physiological maturity of 16 weeks (4 months), this coincides with maximum starch content (73%) for the varieties studied and continued reduction to the 18th week (Figure 1e). Endogenous amylase activity is enhanced in the presence of optimum moisture and temperature conditions. Cured and room‐stored roots (29–31°C and 63–65% relative humidity [RH]) for 4 days (23–26°C and 70–80% RH) exhibited the highest α‐amylase activity for all varieties studied, with the fresh and room‐stored roots showing the lowest activity (Nabubuya, Namuteb, Byaruhanga, Narvhus, & Wicklund, 2012). This therefore implies that the associated changes in starch and sugars of stored roots will be dependent on the state of amylase activity in those roots. Our study showed that starch content for all varieties was generally lowest for the cured and room‐stored roots (47%) and highest for the fresh, room‐stored roots (52%) (Figure 4). This then implies that the amount of native starch required for sweetpotato raw roots will be best selected from 3 to 4 months old fresh and room‐stored roots, where amylase activity is lowest and with highest starch content (Figure 4). For the highest glucose content, sweetpotato roots should be cured and then stored either in the room or in underground pits (Figure 3).

## 4 CONCLUSION

This study has shown the effect of variety on the total starch and individual sugar content of selected Ugandan sweetpotato roots and their variation during root development. It has also highlighted the effect of different postharvest handling and storage conditions on the root carbohydrate content and pasting properties of flour. Storage led to increase in individual sugars and reduction in total starch content which caused reduction in pasting viscosities of the sweetpotato flours. The study also showed that storing of cured roots reduces the peak viscosity of sweetpotato pastes faster than storing of noncured roots hence greatly reducing storage time. Knowledge of these varietal variations and changes during development and under different postharvest handling and storage conditions can be useful in developing models especially in planning the variety, optimum harvest period, and storage conditions and length in order to meet different food industry needs. These findings can therefore be taken into consideration when developing products using sweetpotato flour in the food industry.

## CONFLICT OF INTEREST

Authors declare that there is no conflict of interest.
